# The Editor's Letter-Box

**Published:** 1920-12-11

**Authors:** 


*To the Editor of* The Hospital.

Sir,—I am glad to have had the opportunity of reading Mr. Tennant's letter, but I cannot say that it makes any difference in respect of altering my opinion as stated in the article he criticises. I do not gainsay the influence of faith—no matter what name it may be called by—in helping the body to fight against disease. To deny its value seems to me as illogical as to say that it makes no difference whether we stand up to an enemy or, conversely, turn tail and run away; and almost as illogical as to say that the enemy—microbe, plasmodium, the bullet in the brain—does not exist at all!

Apart from that one postulate—faith—Mr. Tennant's letter seems to me to "cut no ice." It is not possible to deal with it in a paragraph! indeed, it would lead to an endless logomachy of the kind so common in mediæval times. Under various aspects the matter has been debated from a very early stage in our history, and nowadays it is, fortunately, usually confined to the somewhat arid discussions so dear to the "warring sects," and does not infringe upon the valuable space of medical journals.—I am, etc.,

The Writer of the Article.

